# Correction to “Evaluation of a Digital Health Model of Care for the Management of Adults With Symptomatic Malignant Pleural Effusion”

**DOI:** 10.1002/rcr2.70273

**Published:** 2025-07-18

V. Duong, K. Tirant, U. Raza Khan, et al., “Evaluation of a Digital Health Model of Care for the Management of Adults With Symptomatic Malignant Pleural Effusion,” *Respirology Case Reports* 13, no. 6 (2025): e70194, https://doi.org/10.1002/rcr2.70194.

Figures 1, 4, and 5 are incorrect. Below are the corrected figures.

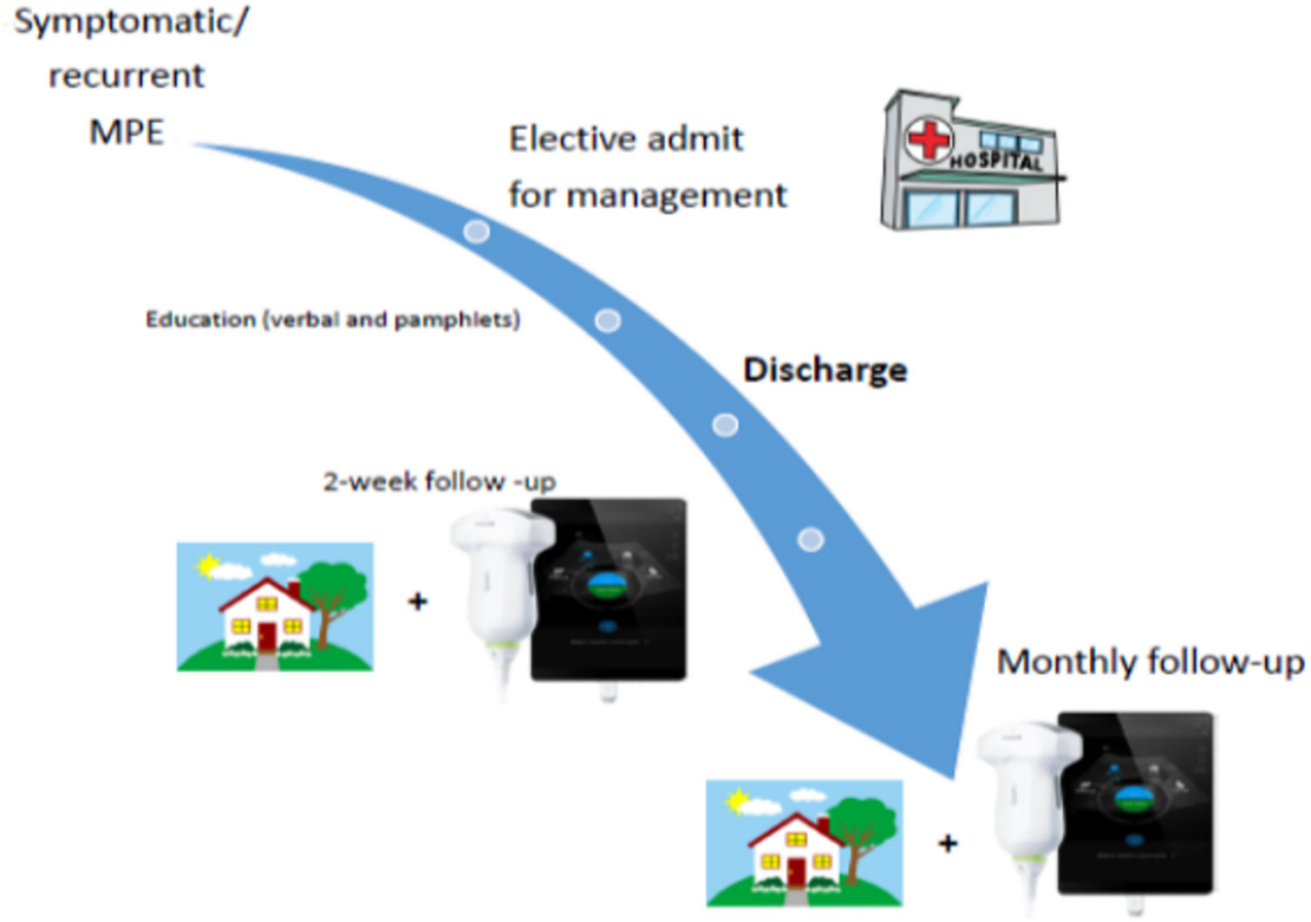

**FIGURE 1** | The proposed SAPS model of care.

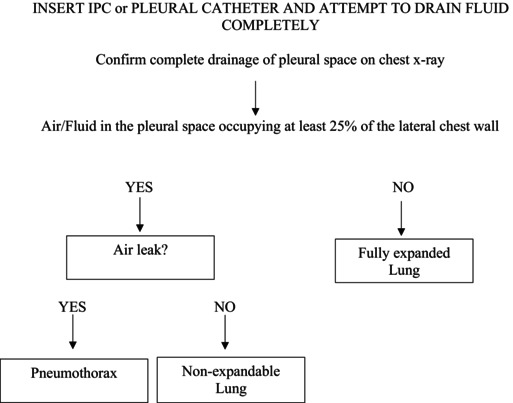

**FIGURE 4** | Standard operating procedure (SOP) for diagnosing non‐expandable lung.

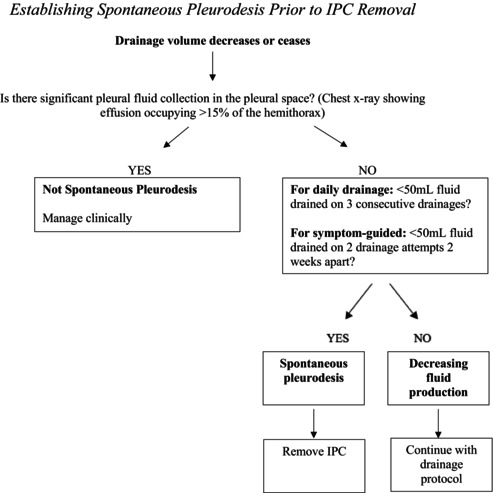

**FIGURE 5** | Standard operating procedure (SOP) for establishing spontaneous pleurodesis in individuals with IPC.

We apologise for these errors.

